# Laparoscopic versus Open Omental Patch Repair for Early Presentation of Perforated Peptic Ulcer: Matched Retrospective Cohort Study

**DOI:** 10.1155/2016/8605039

**Published:** 2016-09-19

**Authors:** Daniel Jin Keat Lee, MaDong Ye, Keith Haozhe Sun, Vishalkumar G. Shelat, Aaryan Koura

**Affiliations:** ^1^Department of Surgery, Khoo Teck Puat Hospital, 90 Yishun Central, Singapore 768828; ^2^Shanghai Medical College, Fudan University, Shanghai 200032, China; ^3^Department of Surgery, Tan Tock Seng Hospital, 11 Jalan Tan Tock Seng, Singapore 308433

## Abstract

*Introduction.* The aim of this study was to compare the outcomes between laparoscopic and open omental patch repair (LOPR versus OR) in patients with similar presentation of perforated peptic ulcer (PPU). The secondary aim was to evaluate the outcomes according to the severity of peritonitis.* Methods.* All patients who underwent omental patch repair at two university-affiliated institutes between January 2010 and December 2014 were reviewed. Matched cohort between LOPR and OR groups was achieved by only including patients that had ulcer perforation <2 cm in size and symptoms occurring <48 hours. Outcome measures were defined in accordance with length of stay (LOS), postoperative complications, and mortality.* Results.* 148 patients met the predefined inclusion criteria with LOPR performed in 40 patients. Outcome measures consistently support laparoscopic approach but only length of hospital stay (LOS) achieved statistical significance (LOPR 4 days versus OR 5 days, *p* < 0.01). In a subgroup analysis of patients with MPI score >21, LOPR is also shown to benefit, particularly resulting in significant shorter LOS (4 days versus 11 days, *p* < 0.01).* Conclusion.* LOPR offers improved short-term outcomes in patients who present within 48 hours and with perforation size <2 cm. LOPR also proved to be more beneficial in high MPI cases.

## 1. Introduction

Laparoscopic omental patch repair (LOPR) of perforated peptic ulcer (PPU) was introduced two decades ago. The earliest prospective studies from Asia successfully demonstrated the safety and feasibility of laparoscopic repair [1, 2]. Though early results were impressive, they were hampered by various shortcomings including selection bias (only reported on patients with uncomplicated ulcers but with high conversion rates of 14–23%), performance bias (experience of surgeons was not well defined), and low statistical power. Studies have argued that reports on LOPR have included patients with early presentation and small perforation size and it remains unclear if LOPR can be applied to all patients [3, 4]. Despite the fact that studies have reported its feasibility, LOPR can be technically challenging for surgeons as it involves steep learning curve and a need for advanced laparoscopic suturing skills. In our local context, due to the emergent nature and after-hour presentation of patients with PPU, most of them would undergo open repair rather than laparoscopic repair [5].

However, a policy of universal laparotomy for all patients in the era of minimally invasive surgery is unjust due to both short-term and long-term morbidities. Laparotomies have been shown to induce a significant physiological stress response which can lead to significant morbidities [6]. Studies have shown that emergency laparotomies are associated with 5% mortality, 20% morbidity, and a 3% long-term risk of bowel obstructions [7]. We believe that a policy of selective use of LOPR would reduce this morbidity and improve perioperative outcomes. Since 2010, there has been an increasing trend towards LOPR for PPU in Singapore. Early experiences have shown that LOPR can be successfully performed when patients present with perforation size <10 mm, located at the pyloroduodenal area, Boey score 0-1, and hemodynamically stable [8].

At present, there is no evidence to suggest that LOPR is a more superior approach compared to open repair (OR). In a recent meta-analysis, Zhou et al. found that laparoscopic repair is slightly advantageous in terms of less postoperative pain and shorter length of stay [9]. However, the authors reckoned that studies included in this analysis, despite being recent and of high quality, lack homogeneous comparison especially among the nonrandomised studies in which they were found to be biased towards selection of younger male patients in LOPR group. We therefore believe that there is a need for comparison between matched cohorts to deliver accurate evaluation pertaining to surgical approach and outcomes.

This current study is an endeavor to compare the short-term outcomes between LOPR and OR only in patients with early presentation of PPU to prevent heterogeneity. The secondary aim is to review postoperative morbidity and mortality between LOPR and OR according to the severity of peritonitis at presentation.

## 2. Methods

The study identified all patients who underwent omental patch repair for PPU in two university-affiliated hospitals between January 2010 to December 2014 and from the Cluster-shared Patient Record System (CPRS). CPRS is a centralized, prospectively maintained database that allows sharing of medical records of patients between public hospitals in Singapore. The clinical and operation records of these patients and a total of 207 patients were analyzed and reviewed. Patients with underlying gastric malignancy who developed a PPU during their hospitalization for other causes and who underwent extended resection (e.g., gastrectomy) or creation of feeding jejunostomies were excluded. Patients were stratified into open repair (OR) and LOPR group. Patients' demographics and perioperative data were obtained from admission notes, operative reports, laboratory results, radiology reports, and discharge summaries. The American Society of Anaesthesiologists (ASA) score, Systemic Inflammatory Response Syndrome (SIRS) criteria, Boey score, and Mannheim Peritonitis Index (MPI) were calculated based on the collected data.

**Figure 1:**
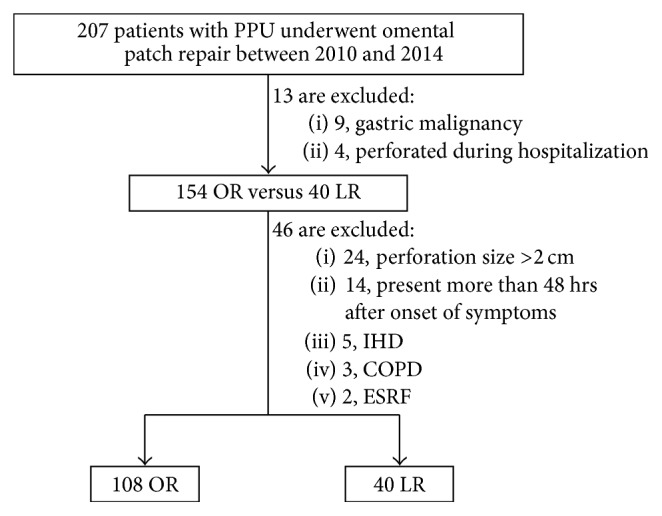
Flowchart of patient selection, inclusion, and exclusion criteria for the study. PPU: perforated peptic ulcer; IHD: ischemic heart disease; COPD: chronic obstructive pulmonary disease; ESRF: end stage renal failure. OR: open repair; LR: laparoscopic repair.

Presently, the decision for laparoscopic versus open repair approach is based on the preference and surgical expertise of the operating surgeons. Our initial analysis revealed that all patients in the LOPR group first presented for medical attention less than 48 hours after the onset of symptoms and had perforations less than 2 cm in size. Furthermore, patients in the laparoscopic group did not have underlying major comorbidities, namely, ischemic heart disease, congestive heart failure, chronic kidney disease, and stroke. To mitigate the biases from these confounders, we decided to exclude patients in the OR groups that presented more than 48 hours after onset of symptoms, with perforation size >2 cm, and those with major comorbidities. This narrowed the data set to 108 patients in the OR group and 40 patients in the LOPR group (Figure 1).

### 2.1. Surgical Procedure

Once the diagnosis of PPU was made, nasogastric tube (NGT) was inserted, an indwelling urinary catheter placed, and broad spectrum antibiotics to cover gut flora was initiated.

All cases of LOPR were performed either by the attending consultants or by trainees under their supervision. LOPR was achieved by using 3- or 4-port technique. In the 4-port technique, the additional port was sometimes used to assist in liver retraction. Once pneumoperitoneum was established, the peritoneal cavity was explored and the degree of contamination was determined. Perforation size was measured approximately in relation to the jaw length of the laparoscopic Maryland dissector (20 mm). The perforation was repaired with a tongue of omentum tied down in place using absorbable 3/0 sutures in interrupted fashion. Intracorporeal knot tying was frequently used. Only 2 surgeons performed extracorporeal knot tying. Peritoneal wash to all 4 quadrants was then performed under direct vision using several litres of warmed saline. The decision for drain placement depended on the degree of peritoneal soilage.

For the OR group, a midline laparotomy incision was used. Following identification of the perforation area, extensive peritoneal toilet was performed using warm saline. Patch repair was then done in standard fashion. Similarly, drain placement was not routine and decided by surgeon's assessment of the degree of contamination. Mass closure of fascia was performed using 1/0 suture and interrupted closure to skin incision subsequently done with either Prolene suture or skin stapler.

### 2.2. Postoperative Management

For the first 24 hours postoperatively, NGT was left to drain with four-hourly manual aspiration. Commencement of feeds was dependent on NGT output and presence of bowel opening signs. All patients began on clear feeds first (such as water, clear fruit juices, and gelatin-based clear jellies) and escalated to full feed (such as cream of rice, strained vegetable juice, and milk) and solid diet as tolerated thereafter.

Therapeutic antibiotics were continued postoperatively. The duration of antibiotic depended on the resolution of infection signs (absence of fever and down trending of inflammatory markers) and resume of bowel movement. Since about 80–90% of juxtapyloric and duodenal ulcers are* H. pylori* associated [10–12], we prophylactically treated these patients postoperatively with* H. pylori* eradication therapy which consisted of amoxicillin and clarithromycin for a total duration of 14 days. Surgical wound was inspected on postoperative day (POD) 3 or upon discharge.

When there were signs of septic complications, patient would undergo a full septic workup consisting of bloods investigation, chest X-ray, and urine analysis. Intra-abdominal sepsis was excluded by computed tomography (CT) scans.

### 2.3. Postoperative Measures

The operation duration was defined as the time from skin incision to the application of wound dressing at the end of the procedure. Relevant postoperative outcomes used for comparison included surgical complications according to Clavien-Dindo (CD) classification [13], wound complications, presence of ileus (defined as daily NG output >500 mLs, nil passing of flatus, or nil commencement of feeds up to POD5), length of intensive care unit (ICU), overall hospital stay, need for repeat operations, and mortality. The length of stay was calculated as the time from admission to discharge, counting the day of admission and operation as day 0.

Analytical tests were performed with IBM SPSS 21. Chi-squared or Fisher's exact test were used for comparison of categorical variable. Student's *t*-test was used to compare continuous variables. Multiple logistic regression analysis was used to assess the association with postoperative complications. *p* < 0.05 was taken as statistically significant.

## 3. Results

Demographics and clinical profiles of patients in each arm are summarized in Table 1. Majority of the patients were male (87.5% were male patients in LOPR group but 89.8% were male patients in OR group; *p* = 0.92). Both groups were comparable in terms of demographic and preoperative physiologic status. There was no conversion from laparoscopic group to open. The only significant differences observed between these two groups were median operation duration (75 min in OR group versus 104 min in LOPR group; *p* < 0.01) and MPI. The LOPR group had significantly higher MPI scores (16 versus 8; *p* < 0.01), indicating a greater degree of peritoneal soilage seen intraoperatively.

Mortality rate in this series is 4.6% (all deaths occur in OR group). 2 elderly patients died due to cardiac events. They died on postoperative days 2 and 16, respectively. A young gentleman at the age of 17 died 6 hours after the operation. He was in shock on admission and had MPI score of 22. Postmortem examination certified death due to severe peritonitis. Another elderly lady at age of 75 years with ASA 3 died on postoperative day 23 due to respiratory failure secondary to hospital acquired pneumonia.

In terms of short-term outcome measure, the LOPR group was associated with significantly shorter length of hospital stay (4 days in LOPR group versus 5 days in OR group; *p* < 0.01). Other outcome measures did not differ significantly between the two groups although results tended to favor the LOPR group. Mortality and incidences of patch leak, reoperation, and life-threatening complications (CD grade 4) were all observed only in the open group.

To evaluate the effect of the severity of peritonitis, we restratified patients according to MPI score of those less than 21 and those with scores of 21 and above. This cut-off value has been previously validated, based on our local case series, as being associated with a higher risk of postoperative complications. In this subgroup analysis (Table 3), outcome measures once again consistently support a laparoscopic approach, but only the shorter length of stays (LOS) was statistically significant (4 days versus 11 days; *p* < 0.01).

Multiple logistic regression analysis to adjust for relevant confounders revealed that LOPR is associated with a shorter length of hospital stay (Table 4).

## 4. Discussion

The major benefits of laparoscopic surgery stem from the requirement of only a few small incisions which would result in improved recovery, better cosmesis, and lesser pain in patients compared to open surgery. The results of the study have demonstrated that the LOPR approach is a feasible, safe option and associated with shorter length of hospital stay for PPU patients with small perforation size presented to hospital in less than 48 hours from the onset of symptoms. These findings correlate with results from several other studies [14–18]. In this study, the difference in length of hospital stay was not affected by the need for rehabilitation postoperatively, as less than 5% of patients subsequently were being discharged to community hospitals/nursing home (Table 2). The study also showed less septic complications, surgical site infections, postoperative ileus, and reoperation rates in the LOPR group. These were also comparable to a recent Cochrane review which concluded similar advantageous outcomes of laparoscopic surgery, although statistically significant differences were not observed [19].

Apart from favorable postoperative outcomes, this study has also shown a significant benefit of adopting the LOPR approach to PPU patients with severe peritonitis. The definition of severe peritonitis in our study is MPI of 21 or greater. This is based on a locally validated study which showed that MPI is the only scoring system which was able to predict all significant morbidities, that is, intra-abdominal collection, leak, reoperation, and mortality [5]. Various studies have also reported the efficacy of MPI as an independent prognostic scoring system in predicting outcome in secondary peritonitis. The cut-off value in these studies ranged between 21 and 26, with sensitivity and specificity of 92–100% and 65–79% [20–22], respectively. The stark difference observed in median LOS in the LOPR group compared to OR group when MPI is >21 (4 days versus 11 days) justified the theoretical advantage and safety of the LOPR approach in PPU patients with severe peritonitis. Laparoscopic surgery has indeed been shown to be associated with lower systemic inflammatory response compared to open surgery [23]. Besides that, laparoscopic approach allows better visualization of the peritoneal cavity and its recesses to facilitate satisfactory washing of the peritoneal cavity compared to an open approach.

This study reported a longer duration of surgery in the LOPR group (104 minutes versus 75 minutes in OR group). Only a few comparative studies have been reported otherwise [24–26]. Longer operating durations seen in the LOPR approach may be associated with the technically more challenging laparoscopic suturing technique. Siu et al. in their earlier experience reported that laparoscopic suture repair is associated with higher conversion to laparotomy when it becomes not feasible in hemodynamically unstable patients, in nonduodenal ulcers, and in larger ulcers (>10 mm size) [27]. In our LOPR group, there were neither any patients of conversion nor leakage observed postoperatively due to suture repair failure. It was found that when intracorporeal suturing was difficult, the extracorporeal technique was employed. To reduce operative time, sutureless repair of PPU has been introduced by using fibrin glue or gelatin plug [28–30]. We are not strong proponents of this approach as it has been shown to be associated with a higher rate of leakage.

The longer operating duration seen in LOPR group is also a result of meticulous peritoneal irrigation which is especially crucial in patients with diffuse peritonitis. Many studies have proven that an adequate peritoneal washout is an independent predictive factor of decreased septic abdominal complications [31–33]. Therefore, as long as patients are hemodynamically stable and adequately resuscitated, an extended duration of laparoscopic operation does not prove to be disadvantageous as demonstrated in this study.

The limitation of this study is a small sample size in the LOPR group. The LOPR for PPU is still not a widely adopted choice of approach even for stable patients with early presentation in our local institutions. This could be attributable to the nature of PPU being a surgical emergency compounded by a lack of availability of senior expertise throughout the day to perform LOPR. We believe that larger sample size in the laparoscopic group will result in an outcome with greater statistical significance.

Despite our study being a retrospective cohort study, the matching performed between the patients in the LOPR and OR groups allowed for an unbiased comparison between both groups. The extensive electronic medical records in our national electronic medical record database enabled us to retrieve all relevant data effectively. We did not analyze certain outcomes, for example, analgesia usage, time to removal of nasogastric tube, time to drain removal, and time to commence diet as these outcomes are vulnerable to reporting and recall bias which would affect the accuracy of any outcome analysis. Essentially, postoperative complications, reintervention rate and the number of days taken for patient to be discharged well, are the ultimate short-term goals of any surgery.

## 5. Conclusion

In this study, we demonstrated that the LOPR for PPU offers improved short-term outcome over the OR approach when these patients present to first medical contact <48 hours from the onset of symptoms and have perforation size of <2 cm. In our subgroup analysis, the LOPR group has also demonstrated a significant benefit in PPU patients with severe peritonitis. We propose that a diagnostic laparoscopy, with a view to proceed with laparoscopic ulcer repair, should be considered as first-line surgical approach for patients with early presentations of PPU.

## Figures and Tables

**Table 1:** Patient demographics.

Characteristics	Open (*n* = 108)	Laparoscopic (*n* = 40)	*p*
Median age (range)	50 (17–92)	47 (20–86)	0.57
Men (%)	97 (89.8%)	35 (87.5%)	0.92
Smoker, *n* (%)	53 (49.1%)	13 (32.5%)	0.07
Alcohol user, *n* (%)	14 (13.0%)	7 (17.5%)	0.48
Admission within 24 hrs after symptoms onset, *n* (%)	90 (83.3%)	38 (95.0%)	0.07
Preoperative lab value			
WBC (k/*μ*L), *n* (%)			0.87
<4 OR >12	61 (56.5%)	22 (55.0%)	
4–12	47 (43.5%)	18 (45.0%)	
Creatinine (*μ*mol/L), *n* (%)			0.30
<130	98 (90.7%)	39 (97.5%)	
*⩾*130	10 (9.3%)	1 (2.5%)	
Hematocrit (%), *n* (%)			0.79
<42	38 (35.2%)	15 (37.5%)	
*⩾*42	70 (64.8%)	25 (62.5%)	
INR, *n* (%)			0.63
*⩾*1.3	7 (6.5%)	1 (2.5%)	
<1.3	94 (87.0%)	36 (90.0%)	
Not done	7 (6.5%)	3 (7.5%)	
ASA class, *n* (%)			0.33
1	23 (21.3%)	13 (32.5%)	
2	52 (48.1%)	18 (45%)	
3	33 (30.5%)	9 (22.5%)	
Mannheim peritonitis index (range)	8 (2–34)	16 (2–26)	<0.01
Mannheim peritonitis index, *n* (%)			0.04
⩽20	94 (87.0%)	28 (70.0%)	
21–29	10 (9.2%)	12 (30.0%)	
>29	4 (3.7%)	0 (0%)	
Shock^#^, *n* (%)	17 (15.7%)	6 (15.0%)	0.91
SIRS^*∗*^, *n* (%)	19 (17.6%)	4 (10.0%)	0.26
Median size of perforation (mm), range	5.0 (2.0–15.0)	5.0 (0.8–15.0)	0.10
Site involved, *n* (%)			0.38
Duodenal	88 (81.5%)	35 (87.5%)	
Gastric	20 (18.5%)	5 (12.5%)	
Median operation duration (min), range	75 (35–175)	104 (65–145)	<0.01

ASA: American Society of Anaesthesiologists. NA: not applicable. WBC: white blood count. SIRS: systemic inflammatory response syndrome.

^#^Shock defined as systolic blood pressure of less than 100 mmHg or heart rate of more than 100 beats per minute.

^*∗*^SIRS defined as 2 or more of the following variables: (1) fever of more than 38°C (100.4°F) or less than 36°C (96.8°F); (2) heart rate of more than 90 beats per minute; (3) respiratory rate of more than 20 breaths per minute or arterial carbon dioxide tension (PaCO_2_) of less than 32 mmHg; (4) abnormal white blood cell count (>12,000/*µ*L or <4,000/*µ*L or >10% immature forms).

**Table 2:** Perioperative comparison between open and laparoscopic repair of perforated peptic ulcer.

Items	Open (*n* = 108)	Laparoscopic (*n* = 40)	*p*
CD classification, *n* (%)			0.34
0-I	93 (86.1%)	37 (92.5%)	
II	4 (3.7%)	2 (5.0%)	
III	1 (0.9%)	1 (2.5%)	
IV	5 (4.6%)	0 (0%)	
V	5 (4.6%)	0 (0%)	
Wound complication, *n* (%)	6 (5.6%)	0 (0%)	NA
Ileus, *n* (%)	2 (1.8%)	2 (5.0%)	0.63
Organ space infection, *n* (%)	5 (4.6%)	1 (2.5%)	0.91
Leakage, *n* (%)	1 (0.9%)	0 (0%)	NA
Ventilation *⩾*48 hrs, *n* (%)	2 (1.8%)	0 (0%)	NA
Sepsis, *n* (%)	5 (4.6%)	0 (0%)	NA
Return to OT, *n* (%)	1 (0.9%)	0 (0%)	NA
Death, *n* (%)	5 (4.6%)	0 (0%)	NA
Median ICU stay (days), range	0 (0–22)	0 (0–5)	0.16
Median hospitalization stay (days), range	5 (3–96)	4 (2–15)	<0.01

CD: Clavien-Dindo. OT: operating theatre. NA: not applicable. ICU: intensive care unit.

**Table 3:** Effects on length of stays and postoperative complications stratified by Manheim peritonitis index total score.

	Open	Laparoscopic	*p*
*Manheim peritonitis index ≤21*			
CD classification, *n* (%)			
0-I	83	27	0.36
II–V	11	1	
Median ICU stay (days), range	0 (0–7)	0 (0–2)	0.45
Median hospitalization stay (days), range	5 (3–96)	4 (2–10)	<0.01

*Manheim peritonitis index >21*			
CD classification, *n* (%)			
0-I	10	11	0.33
II–V	4	1	
Median ICU stay (days), range	0 (0–22)	0 (0–5)	0.55
Median hospitalization stay (days), range	11 (4–28)	4 (3–15)	<0.01

ICU: intensive care unit. CD: Clavien-Dindo.

**Table 4:** Logistic regression analysis of surgical outcomes between laparoscopic and open group with associated variables.

	Crude OR (95% CI)	*p* value	Adjusted OR^#^ (95% CI)	*p* value
CD classification *⩾*II	0.33 (0.07, 1.50)	0.150	0.25 (0.04, 1.55)	0.138
Ileus	4.08 (0.55, 30.32)	0.170	4.45 (0.18, 111.11)	0.363
Organ space infection	0.53 (0.06, 4.66)	0.566	1.84 (0.10, 33.78)	0.680
ICU stay	2.24 (0.77, 6.49)	0.138	3.20 (0.73, 14.09)	0.124
Hospital stay *⩾*6 days	0.37 (0.16, 0.86)	0.022	0.15 (0.04, 0.54)	0.004

OR: odds ratio, CD: Clavien-Dindo.

^#^Adjusted odd ratios were calculated using logistic regression analysis. Analyses were adjusted for age, smoker, ASA class, MPI score, and operation time.
